# Organizational Context and Capabilities for Integrating Care: A Framework for Improvement

**DOI:** 10.5334/ijic.2416

**Published:** 2016-08-31

**Authors:** Jenna M. Evans, Agnes Grudniewicz, G. Ross Baker, Walter P. Wodchis

**Affiliations:** 1 Institute of Health Policy, Management & Evaluation, University of Toronto, CA; Staff Scientist, Cancer Care Ontario, CA; 2 Institute of Health Policy, Management & Evaluation, University of Toronto, CA; Collaboratory for Research & Innovation, Lunenfeld-Tanenbaum Research Institute, Sinai Health System, CA; 3 Institute of Health Policy, Management & Evaluation, University of Toronto, CA; 4 Institute of Health Policy, Management & Evaluation, University of Toronto; Research Scientist, Toronto Rehabilitation Institute; Adjunct Scientist, Institute for Clinical Evaluative Sciences, CA

**Keywords:** integrated care, integrated delivery system, organizational capabilities, organizational context

## Abstract

**Background:**

Interventions aimed at integrating care have become widespread in healthcare; however, there is significant variability in their success. Differences in organizational contexts and associated capabilities may be responsible for some of this variability.

**Purpose:**

This study develops and validates a conceptual framework of organizational capabilities for integrating care, identifies which of these capabilities may be most important, and explores the mechanisms by which they influence integrated care efforts.

**Methods:**

The Context and Capabilities for Integrating Care (CCIC) Framework was developed through a literature review, and revised and validated through interviews with leaders and care providers engaged in integrated care networks in Ontario, Canada. Interviews involved open-ended questions and graphic elicitation. Quantitative content analysis was used to summarize the data.

**Results:**

The CCIC Framework consists of eighteen organizational factors in three categories: Basic Structures, People and Values, and Key Processes. The three most important capabilities shaping the capacity of organizations to implement integrated care interventions include Leadership Approach, Clinician Engagement and Leadership, and Readiness for Change. The majority of hypothesized relationships among organizational capabilities involved Readiness for Change and Partnering, emphasizing the complexity, interrelatedness and importance of these two factors to integrated care efforts.

**Conclusions:**

Organizational leaders can use the framework to determine readiness to integrate care, develop targeted change management strategies, and select appropriate partners with overlapping or complementary profiles on key capabilities. Researchers may use the results to test and refine the proposed framework, with a focus on the hypothesized relationships among organizational capabilities and between organizational capabilities and performance outcomes.

## Introduction

Integrated care interventions promote linkages among diverse professionals and organizations as a means to contain costs, improve quality of care, and enhance the patient experience [1]. Despite the growth of integrated care interventions, there is considerable variability in their success and relatively little is known about what factors are associated with successful implementation across settings [2,3,4]. Scholars recommend tailoring successful integrated care interventions and associated best practices to the local context [2,5,6]. In addition to consideration for the broader social, political, economic and cultural environment [7], policy-makers and leaders must also understand and optimize the context and internal and collective capabilities of the organizations implementing integrated care interventions [2,8]. Studies of integrated care delivery highlight the importance of a range of organizational factors, such as leadership style, organizational culture, resources, information technology, history of change and innovation, organizational bureaucracy, commitment to quality improvement, and patient-centeredness [9,10,11,12]. Differences in these contextual factors and capabilities may be partly responsible for the mixed experiences and performance outcomes described in the literature on integrated care [8,13].

In the field of implementation science, the term “organizational context” is used broadly to describe the setting in which a proposed change is to be implemented [14] and to capture all factors that are not a direct part of the intervention [15]. We frame contextual factors within organizations as “organizational capabilities” in order to convey that these factors are not static features of organizations, but rather dynamic elements subject to growth and decline. We define “organizational capabilities” as the capacity of an organization, or group of organizations, to perform coordinated sets of tasks that support integrated care delivery [16]. Many organizational capabilities, such as governance, leadership, information technology, and partnering are widely recognized as influencing the success or failure of integrated care interventions [11,17,18,19], but the current knowledge base lacks specificity about when and how these factors matter across diverse settings [2,3]. This gap in knowledge is attributable to the limited description and measurement of organizational capabilities in empirical studies on integrated care. Many studies that evaluate integrated care interventions only describe the involved organizations in a few sentences, if at all, [e.g., 20,21,22] or only incorporate easy to measure variables such as the organizations’ age, size and staff mix [23,24,25]). Some integrated care studies use qualitative case study methods to provide more detailed assessments of organizational capabilities [e.g., 26,27], but the organizational capabilities examined vary and there is no common framework.

Both quantitative and qualitative studies of integrated care tend to focus on information technology, inter-professional teamwork, and partnerships, and exclude other key organizational factors such as resources, leadership approach, and internal readiness for change [e.g., 28,29]. Factors such as information technology, inter-professional teamwork and partnerships straddle the boundary between organizational capabilities and the intervention itself. In other words, they reflect the *functionality* of integrated care interventions and the *extent* to which care is integrated. This focus on understanding and measuring progress towards integrated care delivery, as opposed to the underlying organizational capabilities and conditions, is common in the empirical literature on integrated care. However, ongoing challenges to integrating care [e.g., 11,27,30] and expert opinion regarding the importance of adapting integrated care interventions to the local organizational and environmental context [5,6] highlight the potential value of this paper’s focus on understanding and optimizing organizational capabilities for integrating care. Without understanding the organizational capabilities that support integrated care, researchers can face challenges in generalizing findings and best practices across settings, and leaders and care providers can encounter unanticipated barriers to achieving integrated care.

The aim of this study was to develop and validate a comprehensive conceptual framework of organizational capabilities for integrating care, and to explore the mechanisms by which they influence integrated care efforts. Specific objectives of the study were to:

1. Identify and describe organizational and inter-organizational capabilities which influence the implementation, management and sustainability of integrated care interventions;
2. Propose a conceptual framework to collate and organize these factors, and to capture their high-level relationships; and
3. Validate the framework and prioritize the most important organizational capabilities based on the experiences of key informants involved in integrating care.

## Key Concepts and Literature

Few precise definitions of organizational context are evident in the literature. Management scholars describe context as “situational opportunities and constraints” [31] and “organizational characteristics” [32] that exist in the environment surrounding the individual, usually at a higher level of analysis [33]. Johns [31] identifies three dimensions of organizational context: (1) physical context (e.g., built environment), (2) social context (e.g., interaction, information sharing), and (3) task context (e.g., autonomy, resources).

We frame contextual factors within organizations as “organizational capabilities” in order to convey that these factors are not static features of organizations, but rather dynamic elements subject to growth and decline. Organizational capabilities constitute an organization’s *capacity* to perform coordinated sets of tasks [16]. This study focuses on organizational capabilities for integrating care, which refer to the capacity of an organization, or group of organizations, to perform coordinated sets of tasks that support integrated care delivery [16]. This definition encompasses both the internal capabilities of individual organizations as well as the collective inter-organizational capabilities needed to leverage and combine knowledge and resources from multiple organizations in the delivery of integrated care. Organizational capabilities for integrating care derive from the knowledge and skills of the people in the organization as well as organizational structures, processes, and norms supporting (explicitly or implicitly) integrated care [34]. These capabilities enable repeated and reliable performance of an activity, such as integrated care delivery, and develop over time through experience [16].

For most healthcare organizations, integrated care delivery involves multi-level changes to organizational structures, operational activities, and external relationships [1]. This raises the question of how organizational capacity to integrate care (to which organizational capabilities contribute) differs from organizational readiness for the changes involved in integrating care. In his seminal work building a theory of organizational readiness for change, Weiner [35] stresses the importance of defining readiness for change in psychological and social terms, rather than structural terms. In other words, organizational readiness for change focuses on individual and collective attitudes, beliefs and intentions regarding a change, while organizational capacity to implement change refers to the organizational structures and resources needed to implement and sustain change [35,36].

The Resource-Based Theory of the Firm views heterogeneity in resources and capabilities across organizations as an explanatory factor for heterogeneity in performance [13]. Organizations achieve superior performance through resources and capabilities that are valuable, rare, not easily imitated, and/or not substitutable [13,34]. In general, resources are tradable and not unique to the organization, while capabilities are specific to and embedded in the organization and not transferable [13,34]. As such, superior performance is most often achieved through an organization’s capabilities [37].

Our definition of organizational capabilities for integrating care encompasses both structural (tangible) and psycho-social (intangible) features of organizations, consistent with Johns’ [31] tripartite framework of organizational context. The framework we developed thus includes a range of organizational characteristics, including organizational structures, resources, psychological states, social values, and key processes that support integrated care delivery.

## Methods

A two-stage approach, consisting of a literature review and semi-structured interviews, was used to develop the CCIC Framework. The first stage of the study involved reviewing the literature to identify and collate organizational capabilities that support the implementation and delivery of integrated care. Included papers were identified through a previous review of the literature on health systems integration (n = 114 papers) [38]. The review by Evans et al. [38] was selected because, when compared to other recently published reviews of the integrated care literature, it was broader and more inclusive, resulting in a higher number of included papers. This review of the health sciences literature indexed in PubMed and EMBASE between 1985 and 2013 was focused on integration at the systems level, encompassing multiple sectors, organizations and professionals involved in the delivery of healthcare services. Papers focused exclusively on integrating specific programs or services (e.g., mental health), and those reporting on integrating care for a specific population group (e.g., children) were excluded. To supplement this review and identify more recent publications, we conducted a search using the same databases and search terms for papers published after 2013. All relevant papers were included, regardless of methods or methodological quality. Based on our definition of organizational capabilities, we extracted organizational characteristics and activities identified as important from included papers and grouped them together under preliminary categories. The development of the framework was an iterative process, which continued as the review of papers progressed, resulting in minor changes to organization and wording.

Although the CCIC Framework is rooted in the academic literature on integrated care and supported by theory and evidence from multiple disciplines, the extent to which the framework reflects the views and lived experiences of healthcare stakeholders engaged in integrating care was unclear. To validate the framework and help prioritize important factors in practice, semi-structured interviews were conducted with organizational leaders and care providers involved in an integrated care initiative known as “Health Links” in Ontario, Canada. The Health Links are a provincial initiative launched by the Ontario Ministry of Health and Long-Term Care in December 2012 with the aim of integrating care for high needs, high cost patients through voluntary partnerships among health and social services organizations [39]. Organizations interested in partnering to form a Health Link submitted readiness assessments and business plans to the Ministry for approval and received limited development funding. To encourage local innovation, the Ministry adopted a flexible approach to defining and implementing Health Links [39,40]. As such, the Health Links vary widely in terms of their target patient population(s), types of partners, scope of services, and integrated care strategies. Health Links are accountable to regional governing bodies known as Local Health Integration Networks (LHINs); there are fourteen LHINs in the province.

Health Link networks were purposefully sampled to maximize variation in geographic location, implementation stage, and lead organization (each network is led or co-led by a hospital, primary care group, or community-based organization). To identify key informants to participate in the study, we contacted each Health Link’s LHIN for a list of participating organizational leaders and care providers. We invited all leaders and providers suggested by each LHIN to participate in the study. A snowball sampling method was subsequently used to identify additional participants. Multiple participants per organization and Health Link network were invited to participate.

The interviews were divided into two sections. In the first section, we asked participants to describe their general experiences to date with the Health Links initiative. We probed on issues such as successes and challenges, and the factors that contributed to them. We looked for information on internal issues within the organization that may be shaping the performance of the network. We also looked for issues or challenges that are shared across partners. In the second section of the interview, we used a method called ‘graphic elicitation’ [41] where we showed the participants a simplified version of the CCIC Framework and documented their reactions and suggestions. We also asked them to select the factors they considered to be the most important to implementation success and overall performance. Interviews were conducted by JME and AG in person with participants based in the Greater Toronto Area (GTA), and over the phone for those outside of the GTA. The interviews were audio recorded and transcribed verbatim. All interviews took place between October 2014 and February 2015. The study received ethics approval from the Office of Research Ethics at the University of Toronto (protocol reference #29787).

We coded the transcripts deductively based on the CCIC Framework using NVivo software. Codes were also generated inductively for organizational capabilities mentioned by participants and not already reflected in the CCIC Framework. We used quantitative content analysis to identify the frequency with which each factor in the CCIC Framework was mentioned by participants in each section of the interview. We used thematic groups of text as the unit of analysis; as such, several consecutive sentences focused on one factor were counted once. We coded text under a factor in the CCIC framework if it aligned with our definition of that factor. Participants did not have to use the terms in the CCIC Framework. For example, we mapped a description of “organizational glue” to the factor “organizational culture”, a description of “rapid cycle testing” to the factor “quality improvement”, and a description of “organizations working together” to the factor “partnership”. We also documented instances where participants made explicit connections between factors, or described relationships among factors, to identify recurrent patterns across multiple interviews. To ensure consistency, all coding was conducted by JME. AG reviewed coding for the first 30% of the transcripts (7/23); disagreements were resolved by discussion and consensus, and the coding guidelines were updated accordingly, before the remaining transcripts were coded.

## Findings

### Literature Review

Through a synthesis of the extracted data, we identified 17 organizational capabilities that may shape collaborative activities aimed at integrating care. Using these results, we developed a preliminary version of the CCIC Framework with six capabilities categorized under “Basic Structures and Design” (Physical Structures, Human and Material Resources, Organizational Design, Governance, Accountability, and Information Technology), four capabilities categorized under “Leadership and Strategy” (Leadership Approach, Clinician Engagement and Leadership, Strategic Focus on Improvement, and Performance Measurement), three capabilities categorized under “Social and Psychological Context” (Readiness for Change, Organizational Culture, and Work Environment), and four capabilities categorized under “Processes” (Partnering, Teamwork, Delivering Care, and Improving Quality). Expected performance outcomes of integrated care interventions were included in the framework, such as quality of care and patient health status. In addition to these distal outcomes, we included organizational and network capacity to integrate care as a proximal outcome influenced by organizational context and capabilities.

### Semi-Structured Interviews

We emailed 36 invitations for interviews and emailed a follow-up to non-respondents. Participants were welcome to invite colleagues that were also involved in their Health Link network to the interview. A total of 23 interviews were conducted with 29 individuals, 14 from primary care practices or centers (48%), 10 from hospitals (35%) and five from community-based organizations (17%). Over half of participants were administrative leaders and managers (62%), and the remaining were care providers (38%). Participants represented 38 of 54 active Health Link networks (70%) and 13 of 14 LHINs (93%).

Table 1 outlines the frequency with which each factor in the CCIC Framework was mentioned in the semi-structured discussion portion of the interview, and the frequency with which each factor was ranked “most important” in the graphic elicitation portion of the interview. All 17 organizational capabilities in the CCIC Framework were mentioned by participants directly or indirectly during the first half of the interviews (before the framework was shown). In total, nine capabilities emerged as priorities (defined as the top six most frequently mentioned factors in the first and second half of the interviews respectively) (Table 1): Resources and Information Technology from “Basic Structures”; Leadership Approach, Clinician Engagement and Leadership, Patient-Centeredness and Engagement, Organizational/Network Culture, and Readiness for Change from “People and Values”; and Partnering and Delivering Care from “Key Processes.” Only three capabilities made it to the top six in both portions of the interview, suggesting that these capabilities were viewed as playing a more salient role than other capabilities in shaping the implementation and success of the Health Links; these include Leadership Approach, Clinician Engagement and Leadership, and Readiness for Change. All three of these capabilities are based in the “People and Values” domain of the CCIC Framework.

**Table 1:** Organizational Contextual Factors and Capabilities That Most Influence the Implementation and Delivery of Integrated Care: Results of Key Informant Interviews (n = 29).

Semi-Structured Discussion (frequency)	Graphic Elicitation Ranking (frequency)

Partnering (116)	Clinician Engagement & Leadership (16)
Resources (103)	Patient-Centeredness & Engagement (12)
Readiness for Change (78)	Leadership Approach (11)
Clinician Engagement & Leadership (70)	Readiness for Change (9)
Delivering Care (56)	Information Technology (9)
Leadership Approach (51)	Organizational/Network Culture (9)
Patient-Centeredness & Engagement (31)	Resources (8)
Commitment to Learning (30)	Delivering Care (8)
Information Technology (29)	Governance (7)
Measuring Performance (27)	Partnering (4)
Governance (26)	Improving Quality (9)
Physical Features (19)	Measuring Performance (27)
Accountability (18)	Commitment to Learning (2)
Organizational/Network Design (16)	Accountability (2)
Organizational/Network Culture (15)	Physical Features (1)
Improving Quality (9)	Organizational/Network Design (0)
Work Environment (6)	Work Environment (0)

Once shown the framework in the second half of the interview, participants noted that it aligned with their experiences and was a useful tool for considering and assessing organizational capabilities for integrating care. Participants’ experiences and word choice (in the first half of the interview) and their feedback directly on the framework (in the second half of the interview) were used to make modifications to its content, wording and organization to improve clarity. For example, we replaced the headings “Social and Psychological Context” and “Leadership and Strategy” with “People and Values”. We also changed the factors “Strategic Focus on Improvement” to “Commitment to Learning”, “Human and Material Resources” to “Resources”, and “Physical Structures” to “Physical Features of the Organization/Practice.” We moved “Performance Measurement” from “Basic Structures” to “Key Processes” and re-named it “Measuring Performance” to capture participants’ views of it as an ongoing activity. We amalgamated aspects of “Teamwork” under both “Delivering Care” and “Partnering”. We also modified the examples provided with the definition of each factor to better reflect participant experiences. For example, we replaced “use of evidence-based guidelines” under “Delivering Care” with “use of standardized decision support tools”, because participants noted that guidelines tend to be disease-specific and therefore less applicable to the complex patient populations they work with. We added one capability, “Focus on Patient-Centeredness and Engagement”. Finally, we added boxes on “Characteristics of the Integrated Care Intervention” and “Characteristics of the Patient Population” to the framework to better illustrate the mechanisms by which desired outcomes are achieved.

**Figure 1:**
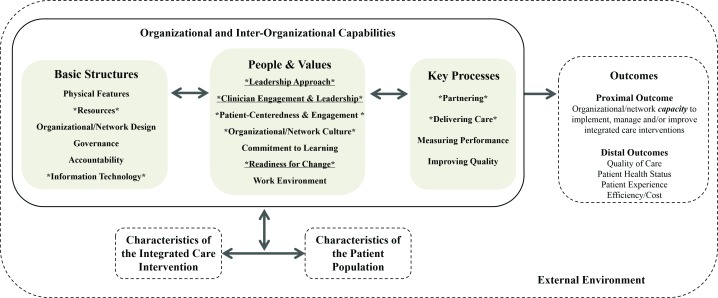
**The Context and Capabilities for Integrating Care (CCIC) Framework.** The CCIC Framework shows how organizational context and capabilities influence the implementation and outcomes of integrated care interventions. Contextual factors and organizational capabilities are organized into three categories: basic structures, people and values, and key processes. These contextual factors and organizational capabilities can be examined within organizations and across partnering organizations in a network. The nine contextual factors and organizational capabilities with the highest frequencies and highest rankings, based on key informant interviews, are depicted using an asterisk (*). The three most important organizational capabilities are underlined and asterisked; these are capabilities which ranked in the top six in *both* the quantitative content analysis of the semi-structured discussion and in the graphic elicitation. Boxes with dashed lines are outside of the scope of this study.

Figure 1 depicts the final version of the CCIC Framework, which consists of eighteen organizational capabilities distributed across three broad categories. The nine organizational capabilities with the highest frequencies and highest rankings in our interviews are depicted using an asterisk (*). The three most important organizational capabilities are underlined and asterisked; these are capabilities ranked in the top six in both the quantitative content analysis of the semi-structured discussion and in the graphic elicitation ranking (Figure 1). Table 2 provides a description and examples for each organizational capability. The definitions were kept intentionally broad to maximize applicability to different types of integrated care interventions.

**Table 2:** Context and Capabilities for Integrating Care (CCIC) Framework: Definitions and Examples.

Concept	Definition	Examples

***Basic Structures***		
Physical Features	Structural and geographic characteristics of the organization/practice and network	organization/practice size and age, urban or rural location, facilities, geographic proximity of network members
Resources	Availability of tangible and intangible assets for ongoing operations at the organization/practice and for network activities	staffing, funding, knowledge, time, project management support, administrative support, brand/reputation
Governance	How the board or steering committee is organized and its activities to direct, manage and monitor the affairs of the organization/practice and network	board/committee composition, types of sub-committees, frequency of meetings, types of decisions made (e.g., extent of centralized planning and standardization)
Accountability	The mechanisms in place to ensure that people and organizations meet formal expectations in the organization/practice and network	regulations enforced by an authority (e.g., government), formal agreements between organizations (e.g., data sharing), organizational mandates, professional scope of practice
Information Technology	The availability and ease of use of technology-based communication and information storage mechanisms in the organization/practice and across the network	shared electronic medical records, email communication, video conferencing, data access and mining, tele-healthcare
Organizational / Network Design	The arrangement of units and roles and how they interact to accomplish tasks in the organization/practice and network	organizational chart (hierarchy), types of departments/programs, job descriptions, communication and decision-making channels (e.g., extent of centralization and formalization)
***People and Values***		
Leadership Approach	The methods and behaviours used by formal leaders in the organization/practice or network (i.e., individual leaders, leadership teams, or lead organizations)	Personal vision for the organization/practice or network, strategies used to empower staff, leadership style and competencies
Clinician Engagement & Leadership	The formal and informal roles held by clinicians in the organization/practice and network, particularly physicians, that enable them to buy-in to and steer change, and influence others	active involvement of clinicians in planning, leading or supporting new initiatives (e.g., clinical champions or directors, networks led by primary care practices)
Organizational / Network Culture*	Widely shared values and habits in the organization/practice or network	perceptions regarding what is important and what is appropriate behavior
Focus on Patient-Centeredness & Engagement	Commitment to placing patients at the center of clinical, organizational and network decision-making	collection and use of patient feedback, consideration for patient needs and preferences, patient input and representation on committees as a standard practice, patient involvement in co-designing services
Commitment to Learning	The existence of a set of values and practices that support ongoing development of new knowledge and insights within the organization/practice and network	experimentation encouraged and rewarded, forums for meeting with and learning from other organizations and external experts, time and resources to reflect on past performance
Work Environment	How employees perceive and experience their job and their workplace in the organization/practice and network	opportunity for input, job satisfaction, burnout
Readiness for Change	The extent to which organizations and individuals are willing and able to implement change in the organization/practice and network	attitudes toward change and toward new or innovative ideas, extent of fit between current vision/strategy and the change
***Key Processes***		
Partnering	The development and management of formal and informal connections between different organizations/practices	sharing information, sharing staff, engaging in collaborative problem-solving, building a common understanding and vision, exchanging knowledge, implementing referral and discharge/transfer agreements
Delivering Care	The methods used by providers in caring for patients in the organization/practice and network	inter-professional teamwork and joint care planning, use of standardized decision support tools, medical model vs. holistic model of care, shared patient-provider decision-making
Measuring Performance	The systematic collection of data about how well the organization/practice and network is meeting its goals	shared performance measurement framework, regular measurement and reporting, data access and mining
Improving Quality	The use of practices and processes that continuously enhance patient care in the organization/practice and network	providing quality improvement (QI) training to staff, systematic use of QI methods (e.g., process mapping, control charts), application of best practices

*Capabilities such as Focus on Patient-Centeredness and Engagement, Commitment to Learning and Readiness for Change may manifest in the culture of the organization or network.

The CCIC Framework suggests that the basic structures, people and values, and processes within organizations and across partnering organizations create a unique local context and set of capabilities that influence the performance and outcomes of integrated care efforts. The framework is explicitly multi-level such that all the capabilities may be examined at the organizational level and/or the network level. Distinct boxes on the patient population and the integrated care intervention are included in the framework to reflect the implementation process as well as the reality of continuous change, whether planned or emergent. A longitudinal, historical approach to conceptualizing and measuring integrated care interventions and associated capabilities aligns with the increasingly popular view of context as a dynamic and fluid process, as opposed to a static state [42]. Although the ultimate outcomes of interest include the patient experience of integrated care as well as performance on various quality and cost indicators, the proximal outcome of interest in the CCIC Framework is the ability and capacity of organizations and networks to carry out collaborative activities aimed at integrating care.

### Relationships among Organizational Contextual Factors and Capabilities

**Figure 2:**
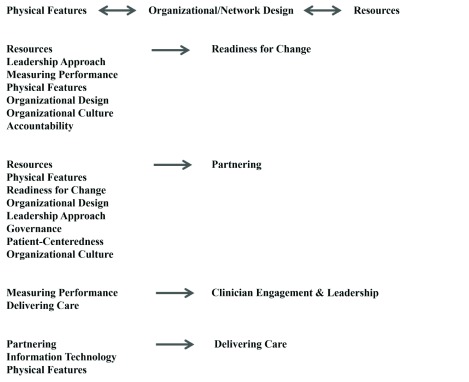
**Most Important Hypothesized Relationships among Organizational Contextual Factors and Capabilities Based on Key Informant Interviews.** The direction of the proposed relationships vary. Some of the relationships are positive, others negative, and some have the potential to be positive or negative, depending on the context and circumstances. Please refer to Supplemental File 1 for details on the proposed relationships.

In their responses to interview questions, participants described relationships among factors in the CCIC Framework (see Supplemental File 1 for a comprehensive list of relationships). The most prominent relationships, based on the number of supporting interviews, are depicted in Figure 2. The majority of hypothesized relationships (23/42 or 55%) involved Readiness for Change and Partnering, emphasizing the complexity, interrelatedness and importance of these two factors to integrated care efforts. Three factors – Resources, Leadership Approach and Organizational Design – were found to influence both Readiness for Change and Partnering. Below, we discuss these three factors and their potential impact on Readiness for Change and Partnering. Sample quotes from participants are provided to help illustrate the proposed relationships.

#### Resources

Some participants highlighted that having more resources, particularly dedicated personnel, helps to maintain confidence and momentum in the change and its sustainability, and that without adequate resources organizations and individuals lack the time needed to engage in and drive the change process. However, others argued that having fewer resources helps people see the need and value of change, and supports both willingness to change and innovative thinking. For example, one participant from a rural Health Link said, “We recognize that the resources that we have are set. We don’t expect the cavalry to come over the hill. So if there are going to be ways and means to change the situation, we’re going to have to work together… I think that’s what drives us to the table” (Interview #15). These differing perspectives on the influence of resources suggest that there may be a curvilinear relationship between resources and readiness for change, and/or that mediating variables may be influencing participant views. For example, prior experience leading or collaborating in change initiatives, and the extent to which these experiences are perceived as positive and successful, may shape how participants view the role and impact of resources.

Resources also influence partnering by shaping the level of trust between organizations and their willingness to collaborate given real or perceived competition for resources. This, in turn, can determine with whom organizations choose to partner. One participant described his experience: “There were more barriers between me and the other Family Health Teams. Not between the providers and me, but between administration and me, where the administration may or may not have perceived us as competing for resources. And where the administration from the other Family Health Teams see that we’re not competing for resources, but in fact, we’re synergistically sharing resources, those barriers have come down” (Interview #18). Organizations with more resources also tend to have more existing relationships that they may leverage to support integrated care.

#### Leadership Approach

Participants highlighted the important role of senior leaders from within partnering organizations. Senior leaders foster interest and commitment to the change and support staff in working differently. Several examples emerged in the interviews in which senior leaders played a pivotal role in changing staff perspectives of perceived barriers to integrated care, such as a lack of resources. One participant in a leadership role said, “It took a year for people to really get it. What people kept saying is, ‘If I could get funding for another nurse practitioner or if I could get this or if I could get that.’ And I kept saying, guys, there’s no more money in the system. This is about working differently” (Interview #6). Another participant commented, “It’s the commitment from leadership from each organization that have said we’re going to realign to make it work. So it’s not that everyone had extra money or capacity lying around. It’s been a leadership choice to realign because they saw the importance of this” (Interview #7).

Senior leaders also influence partnering by shaping the nature and tone of relationship-building processes and by ensuring that the “right people” are meaningfully engaged in the initiative. For example, one participant explained, “That’s why building the relationship is important. If we swooped in and said I’m the lead of this Health Link and you’re going to do this, this and this, we’d never move anything. You come and say, let’s talk about some patient challenges we’re seeing. Do you want to support these patients? And many of these providers are excited because they haven’t been at these tables” (Interview #9).

#### Organizational Design

Participants noted that larger organizations with more hierarchy and formal, centralized decision-making processes are typically less flexible and more difficult to change than smaller, less formal and more decentralized organizations. For example, one participant said, “The other advantage has to do with we are smaller. I mean basically our whole lead team can fit in the same room together. Whereas the larger organizations such as hospitals and CCACs [Community Care Access Centres] don’t have that ability. So we can be a little bit more nimble, a little bit more responsive to change. There’s not as much bureaucracy to go through” (Interview #19).

Such factors also shape partnering. Participants suggested that organizations are often attracted to partners with similar organizational structures, and that factors such as hierarchy, formalization and centralization (and the corresponding level of organizational rigidity or flexibility) can adversely impact information sharing and collaboration among partners. For example, one participant explained, “Family Health Teams tend to be slightly less structured [and have] a little bit more fluidity in how they operate. Versus, for instance, the CCAC [Community Care Access Centre] is slightly more rigidly structured with more set practices and standards. It’s not easy to find ways to collaborate that bridges these ways of operating. There’s a lot of negotiating and trying to learn how to work together in a system that was never really set up well for that collaboration” (Interview #20).

## Discussion

The results of this study provide preliminary validation of the CCIC Framework, which researchers and organizational leaders can use to understand and optimize organizational capabilities influencing the success of integrated care interventions. The quantitative content analysis of participant interviews suggests that the social and psychological context for integrating care should not be neglected in research and practice. Five of the nine organizational capabilities deemed most important to integrating care are from the “People and Values” domain of the framework. An understanding of the subjective context, and associated organizational capabilities such as leadership approach, clinician engagement, organizational culture, and readiness for change, may therefore be of equal, if not greater, importance as study of the objective context such as physical features, resources, and organizational/network design. These findings support Resource-Based Theory, which links superior performance to resources and capabilities that are not easily imitated or substituted [13]. Many of the factors deemed most important by our participants, such as organizational culture, readiness for change, and partnering, are intangible capabilities that were developed over time. These capabilities are embedded in organizations and cannot simply be transferred from one organization to another.

Among the nine prioritized factors, “Leadership Approach,” “Clinician Engagement and Leadership”, and “Readiness for Change” ranked in the top six in *both* the quantitative content analysis of the semi-structured discussion and in the graphic elicitation. “Leadership Approach” and “Clinician Engagement and Leadership” are frequently cited as enablers in the literature on integrated care [11,17,43], but have not been consistently studied. While measures of physician-system integration [e.g., 28] provide insight into the extent of physician economic and administrative involvement in integrated care initiatives, as well as the degree of shared accountability, the extent of engagement and leadership of other providers, such as nurses, is rarely assessed. We are unaware of any studies that examine clinician engagement as a contextual factor (i.e., the extent of clinician engagement and leadership in the organization pre-implementation), rather than as an element or outcome of the integrated care intervention itself.

The study of “Leadership Approach” or leadership style is also limited, with some integrated care instruments measuring the extent of shared leadership [44], team leadership [45,46] or organizational culture, strategic planning, and communication as possible proxies for leadership approach [19,28,47]. While an increasing number of papers on integrated care focus primarily on leadership, the majority are theoretical or discussion papers [48,49,50] with few empirical studies [51].

Qualitative studies of integrated care highlight the importance of readiness for change [11,52,53], but efforts to measure readiness for change and links to the broader literature on organizational change are largely absent in integrated care literature. This study suggests that organizational readiness for change, as it relates to integrating care, may be most influenced by the following factors: resource levels, leadership approach, physical features of the organization, organizational design, organizational culture, accountability structures, and performance measurement (See Supplemental File 1 for an explanation of each proposed relationship). Organizational leaders may have limited control over some of these factors, depending on the timeframe of implementation. However, in our study we identified several examples of organizations modifying their mandate, structures, and processes to enhance readiness for change and support the goals of their Health Link network. Even if such modifications are not possible, awareness of which contextual factors and organizational capabilities most influence readiness to integrate care can inform how leaders design and frame the impending change.

Existing frameworks on organizational change, quality improvement and implementation science recognize the influence of the “organizational context”, “inner setting” or “microsystem” on healthcare transformation efforts [54,55,56,57,58]. However, the CCIC Framework is specific to integrated care; thus, certain elements of prior frameworks take on more importance and are more detailed (e.g., Governance, Accountability, Information Technology, Clinician Engagement and Leadership, Patient-Centeredness and Engagement, Delivering Care, and Improving Quality). The CCIC Framework also encompasses a broader range of organizational-level factors than existing frameworks on the enablers and barriers to integrated care [e.g., 18,43,59,60,61], including Physical Features of the Organization/Practice, Commitment to Learning, and Work Environment, among others. A recent exception is Valentijn et al.’s [62] taxonomy for integrated care, which outlines 59 features across 6 dimensions of integration: clinical, professional, organizational, system, functional and normative. While there is some overlap in organizational factors included in this taxonomy and the CCIC Framework, the frameworks have different aims. The taxonomy aims to clarify the meaning of “integrated care” and to help describe and compare integrated care interventions across multiple dimensions: clinical, professional, organizational, system, functional and normative. The CCIC Framework, on the other hand, aims to help describe and compare the diverse *organizational contexts* within which integrated care interventions are implemented, and to guide organizations and networks in building important capabilities for integrating care. Finally, leaders and clinicians have not always been engaged in developing existing implementation science or integrated care frameworks, meaning their knowledge on what organizational capabilities are important to integrating care is lacking in these frameworks [63]. This may limit their acceptance and use of the frameworks in practice.

Our findings suggest that existing context assessment tools in healthcare may require expansion to measure all organizational capabilities relevant to integrated care. Three notable survey instruments include the Alberta Context Tool [64], the Context Assessment Index [65], and the Survey of Organizational Attributes in Primary Care (SOAPC) [66]. All three instruments measure working relationships and interactions, and to some extent, leadership approach, aspects of organizational culture, and organizational hierarchy. In addition to these factors, the Alberta Context Tool also measures structural and electronic resources, and organizational slack (i.e., time, space and staff) [64]. The Context Assessment Index, on the other hand, also measures patient-provider interactions and respect and use of evidence [65]. Finally, SOAPC also measures history of change, clinician and staff involvement in decision-making, and the extent to which the work environment is stressful [66]. The variation in constructs measured across these instruments attests to the lack of a common framework of contextual factors and organizational capabilities. While most of the factors in the CCIC Framework are measured by one or more of these instruments, the relative emphasis varies with some factors having dedicated scales and others having only one or two relevant items. Researchers and organizational leaders may use validated instruments such as these to inform their understanding of the organizational context for integrating care.

This study has limitations, many of which suggest opportunities for future research. First, the literature review was limited in scope, focusing on system-level integrated care efforts as opposed to those that are population- or sector-specific. While this enhances the applicability to diverse integrated care initiatives of the organizational capabilities identified, relevant capabilities reflected in other sub-sections of the integrated care literature may have been missed. Second, the validation results presented are based on a sample of 29 participants from 38 Health Link networks in one Canadian province. Although the Health Link networks are diverse in terms of geographic context, target populations, supporting structures, organizational partners, and approach to integrating care, the limited scope of the sample indicates that the results may not be widely representative. In particular, the relative importance of each organizational capability may vary based on the integrated care intervention and setting. Almost half of participants represented the primary care sector (48%). This is desirable given that a fundamental aspect of the Health Links model, and one of the key challenges to integrating care in Ontario and elsewhere, is the involvement of primary care. However, the results may also be biased to accounts that focus on primary care. Only 17% of participants represented the community-based sector, even though an increasing proportion of health and social care services are delivered in the community and community-based organizations tend to be more resource constrained than hospitals and primary care. Furthermore, the Health Links initiative was launched in December 2012 and many of the networks in our sample were still in the early stages of implementation and using an incremental approach to change. The results may thus be more applicable to the implementation, rather than the ongoing management and sustainability, of integrated care interventions, and to initiatives implemented using an incremental rather than a rapid approach to change. Other characteristics of the Health Links may also limit the generalizability of the study’s results. For example, many Health Links are governed by formal partnerships and agreements among partners, and receive both financial and in-kind support by the government. Finally, only the perceptions of managers and clinicians were examined. Patient and caregiver perceptions of which organizational capabilities are most important would complement this data.

## Implications for Research and Practice

In this study, we developed a consolidated research- and practice-informed framework to guide the implementation of integrated care interventions and to help focus measurement of organizational context and capabilities. We also prioritized the most important organizational capabilities and explored their inter-relationships via interviews with key informants. Organizational leaders may use the CCIC Framework, and associated data, to inform the implementation of integrated care interventions. For example, the framework can help diagnose strengths and weaknesses within an organization or network. In practice, problems related to organizational capabilities, in particular to the compatibility and alignment (or lack thereof) among partnering organizations, are identified informally and only after considerable time and resources have been invested in implementation [26,67]. An understanding of the key capabilities supporting integrated care delivery can guide early planning to determine “readiness to integrate” and the development of targeted change management strategies that address problem areas or leverage strengths.

Leaders can also use the framework, and associated data, to select appropriate partners with complementary or overlapping profiles on key organizational capabilities. For example, in regards to resources, which include funds, human resources, and knowledge/expertise, diversity and complementarity may be desired. On the other hand, in regards to readiness for change and culture, overlapping profiles may help facilitate the collaborative work needed to achieve integrated care.

The CCIC Framework may be used at various points within the change process, whether during the planning stages as a means of determining readiness to integrate or predicting success, during the implementation process to guide change management efforts, or post-implementation as a means of ongoing evaluation or to determine sustainability. In other words, the framework has potential value throughout the life-cycle of an integrated care intervention as a tool for prospective as well as retrospective reflection and analysis. Integrated care delivery is a long-term endeavor and an open system that evolves over time in response to continuous feedback and learning [48,68,69]. As such, capabilities will change over time [16], whether intentionally or not, necessitating (re-)examination of organizational and network capacity to deliver integrated care. Organizational leaders and researchers may focus on the organizational capabilities deemed most important in this study, or those deemed likely important given the setting, intervention, or patient population. Examining the “lifecycle” of important organizational capabilities as these capabilities move through the founding, development, maturity and branching stages [16] may provide leaders and researchers with a means to understand and strengthen organizational capabilities over time.

Additional research is needed to test and refine the proposed framework, with a focus on the hypothesized relationships among organizational capabilities and between organizational capabilities and integrated care processes and outcomes. Research needs to move beyond general statements about variations in the performance of integrated care interventions being due, for example, to “culture” or “leadership”, to more specific assessments of these capabilities. For example, which aspects of leadership are important at each stage of implementation, by which leaders (e.g., administrative or clinical), and at what level of the organization (senior, middle, or front-line)? To facilitate standardized comparative analysis across interventions and settings, we recommend supplementing qualitative data collection methods with the use of validated survey instruments that measure the concepts in the CCIC Framework, such as those described above. Examining integrated care interventions at various stages of implementation and those that are more and less integrated will enable researchers to draw conclusions about the influence and relative importance of each factor. More detailed analyses of the concepts in the CCIC Framework in future studies may also help focus and refine the concepts, definitions, and examples to improve their relevance and importance to integrated care interventions.

## Supplementary Files

The supplementary file for this article can be found as follows:

- **Supplementary File 1:** Appendix. ijic-16-3-2416-s1.pdf
