# Supplementary material for: Organizational Context and Capabilities for Integrating Care: A Framework for Improvement
Source: Int J Integr Care. 2016 Aug 31;16(3):15. doi: 10.5334/ijic.2416 (PMC5388061; doi:10.5334/ijic.2416)
Supplement: Supplementary file 1 [file ijic-16-3-2416-s1.pdf]

Appendix: Hypothesized Relationships among Organizational Contextual Factors and Capabilities Based on Key Informant Interviews

Related Organizational Capabilities	Description
Basic Structures influence *Basic Structures*	
**Physical Features, Organizational Design and Resources** influence each other	• Larger organizations tend to be more hierarchical and centralized, and have more formal policies and procedures than smaller organizations<br>• Larger organizations also tend to have more staff, administrative support, and slack resources than smaller organizations
**Governance** influences **Accountability**	• Governance structures provide a mechanism for holding individuals and organizations accountable for their activities and performance
Basic Structures influence *People & Values*	
**Physical Features and Organizational Design** influence **Readiness for Change**	• Larger organizations that are more hierarchical and bureaucratic are often less flexible and difficult to change than smaller organizations with less hierarchy and bureaucracy<br>• Smaller organizations tend to be more nimble and responsive to change
**Resources** influence **Readiness for Change**	• More resources helps maintain confidence and momentum in the change and its sustainability<br>• Without adequate resources organizations and individuals lack the time needed to engage in and drive the change process<br>• Fewer resources helps people see the need and value of change towards integrated care delivery, and supports both willingness to change and innovative thinking
**Organizational/Network Design** influences **Work Environment**	• An organization (or network) with a hierarchical and centralized structure for communication and decision-making can reduce staff voice and participation
**Accountability** influences **Readiness for Change**	• Clear lines of accountability (with regards to the integrated care initiative) can help clarify the need for change or act as an incentive for change<br>• Clear lines of accountability can inhibit change and willingness to work together if there is a misalignment between what the organization (or network) is being held accountable for and what the integrated care initiative requires
**Information Technology** influences **Clinician Engagement**	• If clinicians do not perceive that they have the necessary tools (technological and otherwise) to support integrated care delivery, they are more likely to lose interest in the integrated care initiative
**Governance** influences **Commitment to Learning**	• Inter-organizational (and inter-network) committee meetings and events provide a forum for sharing lessons learned and supporting identification and spread of best practices

Basic Structures influence *Key Processes*	
**Physical Features** influence **Partnerships**	• Organizations situated in a rural location tend to have long established relationships with other organizations due to the low number of service providers in the region<br>• The geographic proximity of organizations can influence who an organization partners with as well partnership relations (e.g., the frequency of face-to-face interactions)<br>• Smaller organizations are often perceived as less threatening to organizations looking to partner
**Physical Features** influence **Delivery of Care**	• Geography and geographic proximity of organizations shapes how care is delivered and by whom
**Organizational Design** influences **Partnerships**	• Organizational or network design characteristics, such as hierarchy, bureaucracy, centralization and the associated level of rigidity/flexibility, can limit collaboration and sharing of information<br>• Organizations with a similar organizational design often prefer to partner<br>• Barriers to collaboration that stem from organizational/network design issues may be overcome by strong inter-personal relationships (i.e., people know who to contact for a particular task or problem)
**Governance** influences **Partnerships**	• Inter-organizational governance structures, such as steering committees and boards, provide a forum for bringing partners together
**Resources** influence **Partnerships**	• Real or perceived competition for resources can shape levels of trust among organizations and their willingness to collaborate; it can also shape who an organization chooses to partner with<br>• Resources can shape an organization's (or network's) ability to participate in integrated care initiatives<br>• Larger organizations with more resources often have more existing relationships they can leverage than smaller organizations with fewer resources
**Resources** influence **Delivery of Care, Performance Measurement and Quality Improvement**	• Resources, particularly funds and staff, are required to deliver care to patients, systematically measure performance, and continuously improve quality of care
**Information Technology** influences **Performance Measurement**	• Information technology infrastructure, such as electronic medical records and data access and mining capability, can influence which performance indicators are feasible for use and their quality
**Information Technology** influences **Delivery of Care**	• Information technology tools, such as shared electronic medical records, email communication, teleconferencing, and telehealth, can enhance the efficiency and coordination of care delivery
People & Values influence *People & Values*	
**Leadership Approach** influences **Readiness for Change**	• Leaders can foster interest and commitment to the change and support staff in working differently

**Leadership Approach** influences **Commitment to Learning**	• Leaders can give staff time, space, and resources to reflect on past performance and to brainstorm and experiment
**Leadership Approach** influences **Organizational/Network Culture**	• Leaders can shape which values and habits become engrained in the way the organization (or network) functions; this process occurs over time and is influenced, in part, by how leaders make decisions, the types of decisions they make, what they prioritize as important, and which behaviors they reward or punish
**Organizational/Network Culture** influences **Leadership Approach**	• Leaders who understand the culture of their organization (or network) can tailor their leadership strategies to the culture
**Organizational/Network Culture** influences (or encompasses) **Readiness for Change, Focus on Patient-Centeredness & Engagement, and Commitment to Learning**	• Readiness for change, patient-centeredness and engagement and commitment to learning may be understood as values that may or may not be embedded in the organizational/network culture
**Work Environment** influences **Readiness for Change**	• When staff perceive their work environment to be an open and safe space to share and discuss ideas and concerns, this may contribute to innovative ideas for change as well as change buy-in
**Commitment to Learning** influences **Readiness for Change**	• An organization (or network) that supports learning as a core value and practice is more likely to be open to change
People & Values influence *Basic Structures*	
**Readiness for Change** influences **Organizational Design**	• Organizations that are open to and ready for change are more likely to modify their organizational design to support the integrated care initiative
People & Values influence *Key Processes*	
**Readiness for Change** influences **Partnerships**	• Organizations that are open to and ready for change are more likely to "bend their mandate" and modify structures and processes to support the vision and goal of the partnership<br>• Organizations that are more open to and ready for change may be more likely to partner with one another
**Leadership Approach** influences **Partnerships**	• Leaders shape the nature and tone of relationship-building processes and ensure that the "right people" are meaningfully engaged in the partnership
**Organizational Culture** influences **Partnerships**	• Organizations with similar cultural values are more likely to partner with one another
**Patient-Centeredness & Engagement** influences **Partnerships**	• A focus on patients provides a shared focal point to bring together partners who may have very different mandates and ways of working
**Commitment to Learning** influences	• Organizations that demonstrate commitment to learning are more likely to have effective quality improvement

**Quality Improvement**	efforts
**Patient-Centeredness & Engagement** influences **Performance Measurement**	• An organization-wide (or network-wide) focus on patients informs the types of indicators and strategies used for performance measurement and improvement (e.g., patient satisfaction, patient stories)
Key Processes influence *Key Processes*	
**Delivery of Care** influences **Partnerships**	• When the experiences of clinicians and other front-line staff change or do not change as a result of the integrated care initiative, inter-organizational relations may be affected positively or negatively
**Partnerships** influence **Delivery of Care**	• The types of organizations/practices engaged in the partnership determine which services are included and excluded from the integrated care initiative
Key Processes influence *Basic Structures*	
**Partnerships** influence **Organizational Design**	• The vision and work of the partnership may result in organizations modifying their mandate, structures, and/or processes to support the goals of the partnership
**Performance Measurement** influences **Accountability**	• The collection and reporting of performance data is a mechanism for holding individuals and organizations accountable for their activities and performance
Key Processes influence *People & Values*	
**Performance Measurement** influences **Readiness for Change**	• Positive performance data provides evidence of impact and can therefore help maintain interest and commitment in the change<br>• Performance measurement could be a barrier to change if the focus is on what can be measured and not necessarily on what is most important
**Performance Measurement** influences **Clinician Engagement**	• Performance data shapes the extent to which clinicians are committed to the integrated care initiative; evidence of positive impact results in improved clinician engagement
**Delivery of Care** influences **Clinician Engagement**	• When the experiences of clinicians change or do not change as a result of the integrated care initiative, their level of engagement in the initiative is affected
**Quality Improvement** influences **Commitment to Learning**	• Organizations (or networks) that have a system in place to support quality improvement activities are more likely to exhibit commitment to learning more broadly (i.e., not just in terms of clinical services, but also in regards to management and administration)
